# Help-seeking behaviors among survivors of intimate partner violence during pregnancy in 54 LMICs

**DOI:** 10.1093/eurpub/ckac129.056

**Published:** 2022-10-25

**Authors:** M Stiller, ML Wilson, T Bärnighausen, AA Adedimeji, ER Lewis, A Abio

**Affiliations:** 1 Heidelberg Institute of Global Health, Heidelberg University Hospital, Heidelberg, Germany; 2 Injury Epidemiology & Prevention Research Group, Turku Brain Injury Center, Turku, Finland; 3 Department of Epidemiology & Population Health, Albert Einstein College of Medicine, New York, USA; 4 Department of Surgery, Jacobi Medical Center, New York, USA

## Abstract

**Background:**

Intimate partner violence (IPV) experienced by pregnant women is pervasive worldwide. As survivors rarely seek help, there exists a paucity of research on their help-seeking behaviors. The present study provides a multi-national perspective into the nature of help-seeking behaviors among survivors of IPV during pregnancy.

**Methods:**

Population-based data from 54 LMICs were abstracted from the Demographic and Health Surveys Program between 2005 and 2020 (N = 359,027). Bivariate and multivariable logistic regression were used to analyze the extent to which - and from whom - survivors of IPV during pregnancy sought help and assess associated factors.

**Results:**

Half of respondents (51.87%) sought help following IPV while pregnant (ranging from 39.02% in Asia and Oceania to 63.18% in sub-Saharan Africa). Support was primarily obtained from informal contacts (44.02%), such as family, neighbors, and friends, and rarely from formal institutions (10.45%), such as law enforcement, social and medical services. Help-seeking behaviors were positively associated with higher education, employment, earnings exceeding that of their spouse, exposure to mass media, intimate partner's alcohol consumption, fear of their intimate partner, parental violence, richer wealth status, partner's controlling behaviors, and facing barriers to access health care. Conversely, being married, and justifying wife beating were negatively associated with help-seeking.

**Conclusions:**

The research findings highlight the need for interventions, ranging in scope from the individual to familial and societal levels, to increase and improve help-seeking opportunities for IPV survivors. Efforts should be made on strengthening women's decision-making capacity, reducing poverty, ensuring educational attainment, improving employment opportunities, disseminating information about help sources, IPV screening within health care, and promoting the diffusion of gender equality by engaging communities as a whole.

**Key messages:**

• IPV during pregnancy is still pervasive in LMICs, and only half of survivors seek help. Women’s individual, partner’s/family’s, and community’s factors are associated with IPV survivors’ help-seeking.

• The research findings highlight the need for interventions, ranging in scope from the individual to familial and societal levels, to increase and improve help-seeking opportunities for IPV survivors.

